# The effect of vitreomacular interface in neovascular age-related macular degeneration treated with intravitreal injection of anti-VEGF

**DOI:** 10.1186/s12886-022-02640-3

**Published:** 2022-11-03

**Authors:** Fangyuan Han, Xingwang Chen, Ruyi Zhao, Xin Jin, Wei Tan, Ying Zhang

**Affiliations:** 1 Department of Ophthalmology, The First People’s Hospital of Zunyi (the Third Affiliated Hospital of Zunyi Medical University), 98 Fenghuang North Road, Huichuan District, Zunyi, Guizhou Province 563000 China; 2 Department of Ophthalmology, The Affiliated Hospital of Zunyi Medical University, Zunyi, China; 3 Special Key Laboratory of Ocular Diseases of Guizhou Province, Zunyi Medical University, Zunyi, China

**Keywords:** Vitreomacular interface, Intravitreal injection, anti-VEGF, Age-related macular degeneration

## Abstract

**Background:**

The purpose of this study is to study the effect of repeated intravitreal injection of anti-vascular endothelial growth factor (anti-VEGF) drugs on vitreomacular interface.

**Methods:**

Neovascular age-related macular degeneration patients who received intravitreal injections of anti-VEGF drugs were included. Eyes with severe vitreous opacity, uveitis, complicated cataract surgery and previous vitrectomy were excluded. Vitreomacular interface, best corrected visual acuity (BCVA) and central retinal thickness (CRT) assessment were performed once a month for at least 3 months. The nature and time of the change event are recorded. Groups were divided according to whether vitreomacular interface change events occurred. To analyse the risk factors of vitreomacular interface changes and their influence on treatment effect.

**Results:**

A total of 87 eyes were evaluated. Vitreomacular interface change event occurred in 9 eyes. Pre-existing vitreomacular interface abnormality (VMIA) was a risk factor for the VMI change (*P* = 0.033, OR = 16.518, 95% CI: 1.258 to 216.939). 60% of interface events occurred in the first 3 months of treatment. The final BCVA of eyes with vitreomacular interface unchanged was significantly higher than that at baseline (*P* = 0.001), and the final CRT was also significantly lower than that at baseline (*P* < 0.001). The final CRT of eyes vitreomacular interface changed was significantly lower than that at baseline (*P* = 0.015), however, there was no statistical significance in BCVA (*P* = 0.468).

**Conclusion:**

Intravitreal injection of anti-VEGF drugs has a certain probability to cause changes in the vitreomacular interface, and the risk is higher in eyes with pre-existing vitreomacular interface abnormality. The effect of intravitreal injections on the vitreomacular interface was concentrated in the first three injections, and subsequent increases in the number of injections did not significantly increase the risk of vitreomacular interface abnormality. Ophthalmologists should increase attention to the vitreomacular interface in the early stages of anti-VEGF therapy and counsel patients accordingly.

## Introduction

Since 2006, intravitreal injection of anti-vascular endothelial growth factor (anti-VEGF) drugs has gradually been used for the treatment of neovascular age-related macular degeneration (ARMD) [1]. Nowadays, anti-VEGF drugs have become the first-line treatment of neovascular ARMD [2]. During the course of treatment, repeated injections are usually required to enable patients to obtain better vision [3]. In the real-world study, the average number of intravitreal injections was 6.4 ± 2.4 for ranibizumab and 6.2 ± 2.4 for aflibercept [4]. Therefore, ocular complications caused by repeated intravitreal injections have become an important issue of widespread concern.

Vitreomacular interface abnormalities (VMIA) such as epiretinal membrane (ERM) or vitreomacular traction (VMT) are one of the most common complications of eye surgery. It is well known that ocular surgeries such as cataract extraction [5], retinal detachment reattachment surgery [6], retinal laser photocoagulation [7] and retinal cryopexy [8] are all associated with an increased incidence of ERM formation. As an ocular invasive procedure, repeated vitreous injection of anti-VEGF drugs may also have an adverse effect on the formation of VMIA after a long time. Therefore, increasing attention to ocular complications has become an important issue for anti-VEGF therapy.

At present, the discussion on the relationship between VMIA and ARMD is mainly carried out on the premise of taking the interface as the influencing factor. Vitreomacular adhesions (VMA) can induce the pathogenesis of ARMD through inflammation [9], and VMIA can reduce the response to anti-VEGF agents and lead to an increase in the number of injections [10]. However, the effect of intravitreal injection of anti-VEGF drugs on the vitreomacular interface (VMI) has rarely been discussed.

In this study, we retrospectively investigated the incidence of VMI changes in a cohort of neovascular ARMD patients treated with intravitreal anti-VEGF drugs for at least 3 months. The effect of VMI changes on the efficacy of anti-VEGF therapy in neovascular ARMD was analysed. Systemic and ocular factors associated with changes in the VMI were also analysed. To understand the effects of repeated intravitreal injections of anti-VEGF agents on the VMI and its implications for patient management.

## Materials and methods

### Participants

We conducted a retrospective study of neovascular ARMD patients who received intravitreal injection anti-VEGF therapy at the first people’s hospital of Zunyi from January 2018 to December 2021. Institutional Review Board/Ethics Committee approval and informed consent were obtained for this study. The inclusion criteria were neovascular ARMD diagnosed by fundus fluorescein angiography (FFA), receiving three or more intravitreal injections of anti-VEGF drugs, and follow-up for more than three months. Exclusion criteria were previous vitrectomy, complicated cataract surgery, and eyes with other conditions that are known to affect the VMI, such as uveitis, retinal vascular disease, severe vitreous opacity and diabetic retinopathy.

### Treatment protocol

All intravitreal injections were performed in the operating room using a 30-gauge needle in strict accordance with the aseptic technique. All patients received 3 intravitreal injections of anti-VEGF drugs, 1 month apart. If the best corrected visual acuity (BCVA) or fluorescein leakage worsens again after the third injection, the injection is repeated at the discretion of the treating physician. Intravitreal drugs used in this study included ranibizumab (Lucentis, Novartis), and conbercept (Chengdu Kanghong Biotech, Inc., Chengdu, Sichuan, China). The injected volume of VEGF inhibitors was 0.05 ml for ranibizumab and conbercept.

### Data collection

The gender, age, basic diseases, and lens status of the affected eye were registered at baseline. BCVA, slit lamp, fundus, intraocular pressure and optical coherence tomography (OCT) were examined at baseline and each follow-up. And the FFA examinations were performed at baseline and 1 month after the third injection. The BCVA was performed with a logarithm of the minimal angle of the resolution chart. The values were expressed as mean ± standard deviation. The data of central retinal thickness (CRT) and VMI were measured and evaluated according to the OCT images of each follow-up. Finally, the follow-up period and injection times of each eye were recorded.

The VMI change event was defined as the development of a VMI change in eyes previously normal, progression of an existing interface change, or appearance of a different VMI change in the eyes with a previously existing change. The VMIAs such as Vitreomacular adhesion (VMA), VMT, macular hole and posterior vitreous detachment (PVD) were defined as per standard classification scheme by the International Vitreomacular Traction Study Group classification [11, 12]. Groups were divided according to whether VMI change events occurred.

### Statistical analysis

Quantitative data were expressed as mean ± SD. Differences in quantitative variables between the two groups were analysed using the t-test. BCVA and CRT before and after treatment were compared by paired t-test. The Chi-square test (including Fisher’s exact test if necessary) was used for the comparison of categorical variables. Multivariate logistic regression was applied to those parameters that might be associated with the development of post-injection vitreoretinal interface changes. These parameters included age, hypertension, diabetes, cataract surgery, baseline VMI, baseline BCVA, baseline CRT, anti-VEGF drugs, injection times before VMI change and treatment time. SPSS 18.0 software (IBM, Inc., Chicago, IL, USA) was used. The tests were two-tailed and statistical significance was set at a *P*-value of < 0.05.

## Results

A total of 79 patients (98 eyes) received anti-VEGF therapy for neovascular ARMD at our institution from January 2018 to December 2021, of whom 72 patients (87 eyes) met the inclusion criteria and were included in this study. The clinical characteristics of the patients are shown in Table 1.

**Table 1:** The clinical characteristics of the patients

Characteristics	No. (%) or mean ± SD
Cases (eyes)	72 (87)
Gender	
Male	37 (51.4)
Female	35 (48.6)
Age (years)	71.50 ± 9.11
Hypertension	34 (47.2)
Diabetes	11 (15.3)
Cataract surgery	21 (24.1)
Baseline VMIA	43 (49.4)
VMA broad	14 (16.1)
VMA focal	15 (17.2)
ERM	14 (16.1)
Anti-VEGF	
Conbercept	58 (66.7)
Ranibizumab	29 (33.3)
Total injection times	4.41 ± 2.60
Follow-up (months)	9.09 ± 8.07
Baseline BCVA (Log MAR)	1.01 ± 0.44
Final BCVA (Log MAR)	0.87 ± 0.44
Baseline CRT(µm)	447.11 ± 198.65
Final CRT(µm)	316.06 ± 198.24
Total incidence of VMI change	9 (10.3)
First injection to VMI change (months)	4.00 ± 3.64

Nine of these 87 eyes developed VMI changes during repeated intravitreal injections. The eyes were grouped according to whether VMI changes occurred during the repeated intravitreal injection. At baseline, the proportion of VMIA in VMI changed eyes (88.9%) was significantly higher than that in VMI changed eyes (44.9%, *P* = 0.012). And there was no significant difference in other clinical characteristics between the two groups at baseline, as shown in Table 2. Logistic regression analysis showed that baseline VMIA was positively associated with the change of VMA (*P* = 0.033, OR = 16.518, 95% CI: 1.258 to 216.939), and age, gender, etc. were not associated with the change of VMA (*P* > 0.05), Table 3. All 9 eyes received 1 to 3 intravitreal injections before the change in VMI. Six of the 9 eyes experienced VMI changes within 3 months after the first injection. Details of the changes in VMI in these 9 eyes are shown in Table 4.

**Table 2:** Comparison between patients with VMI changed and unchanged at baseline

Characteristics	VMI changed	VMI unchanged	*P*-value
Male/Female	5/4	32/31	0.789
Age (years)	76.11 ± 7.39	70.84 ± 9.20	0.105
Hypertension (yes/no)	3/6	31/32	0.372
Diabetes (yes/no)	1/8	10/53	0.710
Cataract surgery (yes/no)	3/6	18/60	0.496
Baseline VMIA (yes/no)	8/1	35/43	0.012
Baseline BCVA (Log MAR)	0.95 ± 0.32	1.02 ± 0.45	0.658
Baseline CRT(µm)	473.00 ± 202.45	444.13 ± 199.32	0.682
Conbercept/ Ranibizumab	6/3	52/26	1.000

**Table 3:** Logistic regression analysis of the influencing factors of VMA changes

Variable	*P*-value	OR	CI (95%)	
Gender	0.830	0.818	0.130	5.136
Age	0.217	1.106	0.943	1.297
Hypertension	0.598	0.589	0.082	4.215
Diabetes	0.420	0.329	0.022	4.916
Cataract surgery	0.283	4.000	0.319	50.228
Baseline VMIA	0.033	16.518	1.258	216.939
Baseline BCVA	0.237	0.184	0.011	3.037
Baseline CRT	0.337	1.003	0.997	1.008
Anti-VEGF drugs	0.928	1.098	0.144	8.382
Injection times before VMI change	0.546	1.333	0.524	3.389
Treatment time	0.068	0.750	0.551	1.021

**Table 4:** Details of VMI changes

No.	Baseline VMI	Change type	injections before change (n)	First injection to change (months)
4	ERM	Thickened	2	2
10	ERM	Thickened	3	4
32	VMA focal	PVD	1	1
36	VMA focal	PVD	3	3
49	VMA focal	PVD	2	2
52	VMA broad	ERM	3	12
56	VMA focal	PVD	2	2
71	VMA broad	VMA focal	3	8
78	VMA broad	VMA focal	2	2

**Fig. 1:**
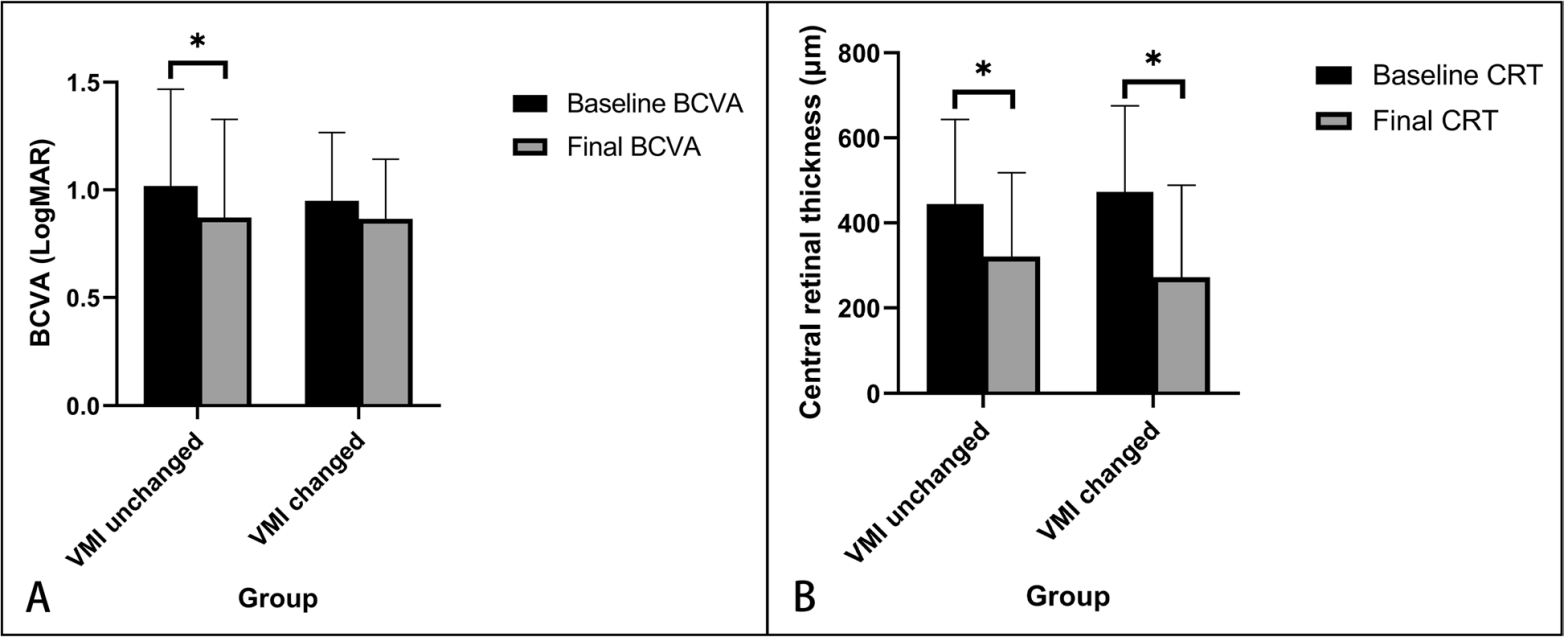
Treatment effect of anti-VEGF in VMI changed group and unchanged group. **A**, The baseline BCVA and final BCVA in eyes with and without VMI changed. **B**, The baseline CRT and final CRT in eyes with and without VMI changed (*P < 0.05)

BCVA and CRT before and after anti-VEGF treatment were compared in the two groups separately. In eyes with VMI unchanged, the final BCVA was significantly higher than that at baseline (*P* = 0.001), and the final CRT was also significantly lower than that at baseline (*P* < 0.001). In eyes with VMI changed, the final CRT was significantly lower than that at baseline (*P* = 0.015), however, there was no significant difference in BCVA (*P* = 0.468). As shown in Fig. 1. In addition, there was no significant difference in the final number of intravitreal injections between the VMI changed group (3.67 ± 1.12) and the VMI unchanged group (4.50 ± 2.71, *P* = 0.366).

## Discussion

Currently, intravitreal injection of anti-VEGF drugs is the standard treatment for neovascular ARMD. Patients with neovascular ARMD require long-term repeated intravitreal injections during anti-VEGF therapy [4, 13]. Repeated injections may lead to complications such as uveitis, endophthalmitis, subconjunctival haemorrhage, elevated intraocular pressure, retinal detachment, retinal pigment epithelium tears, corneal nerve fibres and sensitivity loss [14, 15]. As a common complication of ocular surgery, VMIA has been discussed as an influencing factor of anti-VEGF efficacy in previous studies [10]. To assess the interrelationship between the intravitreal injection therapy and the development of VMIA, this study analysed a cohort of ARMD eyes receiving anti-VEGF therapy. In our results, 49.4% of ARMD eyes at baseline had presented any VMIA and these were roughly equivalent to what Leuschen et al. reported [16]. This prevalence is higher than that in controls with adjusted age. VMIA was considered to be pathogenesis of ARMD, as traction would lead to pigment epithelial detachment and spread of VEGF [17].

In our study, 10.3% of the eyes had the occurrence or development of VMIA after receiving anti-VEGF treatment, which was lower than Chang et al. [18] and Kinra et al. [19]. These two studies involved different underlying diseases, such as diabetic macular edema and retinal vein occlusion. Diabetic retinopathy and retinal vein occlusion mainly involve the inner retinal structure, the VEGF and inflammatory factors are more likely to affect the VMI. Therefore, the response of VMI to VEGF treatment is different in different clinical entities.

In this study, 13.3% of VMA eventually developed PVD, which is higher than the 5.6% incidence of PVD reported by Veloso et al. [20]. The difference may be due to the small sample size. PVD occurred at very similar time points in both studies, both within three injections. Although PVD can occur spontaneously due to its natural history, the fact that PVD occurred in all eyes in these studies within a relatively short time after intravitreal injection suggests that PVD in these cases may be caused by intravitreal injection rather than coincidence. Therefore, we speculate that intravitreal injection may lead to PVD due to its mechanical effect.

It was reported that the coexistence rate of ERM accounts for 15–38% of ARMD eyes [21], which is consistent with 14.9% in our group. The incidence rate of ERM in ARMD eyes was higher than that in normal eyes. This was thought to be due to inflammation or preretinal glial cells in ARMD than control [22]. Previous studies believe that PVD was significantly associated with the formation of ERM [23]. However, in our study, the original ERM was significantly thickened in 2 eyes after intravitreal injection, and 1 eye with VMA developed ERM. It seems that VMIA contributes more to the development of ERM. Some researchers believe that chronic vitreous traction caused the migration of glial cells, macrophages, or pigment epithelial cells, and leads to ERM, which further supports our findings [24, 25].

Regarding the risk factors of VMI changes in patients treated with intravitreal injection of anti-VEGF drugs, in the report of Kinra et al. [19], VMIA, cataract extraction and age are the influencing factors of VMI changes. Stallman et al. observed differences between sexes with respect to the induction of PVD [26]. Chang et al. observed that poor BCVA was an important factor associated with the development of VMIA [18]. However, we did not find a correlation between cataract extraction, age, sex, BCVA, and the development of VMIA in ARMD eyes treated with intravitreal injection. In our study, pre-existing VMIA was an independent risk factor for post-injection VMI changes. Possible reasons for this phenomenon: Some VMAs are a staged state in the natural process of PVD, the perturbation during injection and the destruction of the vitreous gel collagen framework by the injection fluid accelerate the development of PVD [26]; The physicochemical effects of drugs lead to immune and inflammatory responses that stimulate the migration of glial cells, macrophages or pigment epithelial cells caused by vitreous traction [24], which ultimately accelerates the occurrence and development of ERM.

This study showed that the CRT of the VMI changed group and the VMI unchanged group significantly decreased after anti-VEGF treatment, indicating that the anti-VEGF treatment was effective in both groups. In addition, the number of intravitreal injections received by the two groups of patients was similar, and the effect of VMIA on the treatment effect was not obvious. However, previous studies have shown that VMT decreased the response to anti-VEGF agents [10]. We speculate that the effect of VMA and ERM on the treatment effect is relatively limited due to the absence of macular traction. And PVD is beneficial to the treatment [20]. Individually, in eyes where the occurrence or development of ERM was found, CRT thickening and decreased visual acuity were also found. On the contrary, in the eyes of PVD occurred, indicators were improved. Therefore, the impact of different VMIA types on the treatment effect needs to be subdivided. Although BCVA was improved in both groups, there was no significant difference in BCVA before and after treatment in the VMI changed group. This may also be due to the reasons mentioned above.

The main limitation of our study is that there was no control of the eyes with ARMD and without any injection, and its retrospective nature. It is necessary to conduct further prospective large sample research on these parameters.

In conclusion, intravitreal injection of anti-VEGF drugs has a certain probability to cause changes in VMI, and the risk is higher in eyes with pre-existing VMIA. The effect of intravitreal injections on VMI was concentrated in the first three injections, and subsequent increases in the number of injections did not significantly increase the risk of VMIA.

## Data Availability

The datasets generated and analysed during the current study are not publicly available because we are not able to permit any possibility of identifying persons from treatment history regardless of data anonymity, but data are available from the corresponding author upon reasonable request.

## Abbreviations

ARMD: age-related macular degeneration
BCVA: best corrected visual acuity
CRT: central retinal thickness
ERM: epiretinal membrane
FFA: fundus fluorescein angiography
OCT: optical coherence tomography
PVD: posterior vitreous detachment
VEGF: vascular endothelial growth factor
VMI: vitreomacular interface
VMIA: vitreomacular interface abnormalities
VMT: vitreomacular traction
